# Polyaspartate-Ionene/Na^+^-Montmorillonite Nanocomposites as Novel Adsorbent for Anionic Dye; Effect of Ionene Structure

**DOI:** 10.3390/polym12122843

**Published:** 2020-11-29

**Authors:** Hany El-Hamshary, Abeer S. Elsherbiny, Mohamed H. El-Newehy, Mohamed E. EL-Hefnawy

**Affiliations:** 1 Department of Chemistry, College of Science, King Saud University, Riyadh 11451, Saudi Arabia; 2 Department of Chemistry, Faculty of Science, Tanta University, Tanta 31527, Egypt; malhefnawy@kau.edu.sa; 3 Department of Chemistry, Rabigh College of Arts and Sciences, King Abdulaziz University, Jeddah 21911, Saudi Arabia

**Keywords:** poly(succinimide-*co*-aspartate), sodium montmorillonite, nanocomposites, ionene, adsorption, anionic dyes

## Abstract

Surface modification of sodium montmorillonite (Na^+^-Mt) was performed using antimicrobial agents to produce an ecofriendly nanocomposite. The adsorption performance of the nanocomposite has been evaluated for the removal of Acid Blue 25 dye (AB25) as a model organic pollutant from wastewater. Sodium montmorillonite (Na^+^-Mt) was modified with three different ionene compounds through ion exchange, and further modified through reaction with polyaspartate to provide three ecofriendly nanocomposites (denoted ICP-1–3). The nanocomposites were characterized using FTIR, PXRD, TEM, SEM, and BET surface area. The adsorption isotherm of AB25 onto ICP-1, ICP-2 and ICP-3 was analyzed using the Langmuir, Freundlich, and Dubinin–Radushkevich (D–R) models. The adsorption isotherm was found to be best fitted by a Freundlich model. The thermodynamic parameters were calculated. The kinetics of the adsorption data were analyzed and the adsorption behavior was found to obey pseudo-second-order kinetics, and the intraparticle diffusion model. The adsorption mechanism was studied by FTIR.

## 1. Introduction

The wastewater from industrial practices include organic discharges such as textile dyes, printing inks, phenols, pesticides, etc. The presence of small concentrations of dyes can visually be detected, and severely harms aquatic life and food webs and cause other adverse reactions for humans [1,2,3,4]. Although dyes are used in several industries such as food coloring, textiles and painting, they have an adverse effect on the environment. Water pollution is one of the major and most negative effects of using dyes. For example, textile dyes, when discharged into waterways, cause damage to the quality of water bodies, as the presence of residue from these dyes increases the demand for biochemical and chemical oxygen (BOD), as they impede the processes of photosynthesis, which results in preventing plant growth. Eating dependent on a plant contaminated with these dyes will inevitably find its way into the food chain and thus lead to a bio-accumulation that may result in enhanced toxicity and mutagenesis, and perhaps worse, such as carcinogenesis [5]. Therefore, it has been important to search for effective procedures to remove organic dyes from wastewater. Currently, methods used for dye removal include: membrane filtration, [6] ion exchange, [7,8] chemical precipitation, [9] and adsorption [7,10,11]. Among these procedures, adsorption onto natural materials is preferred due to its low cost. The adsorptive properties of naturally occurring substances including activated charcoal, agricultural residues, polysaccharides, peat, and fungal and bacterial biomass have all been examined, [12] in addition to nanoclays and zeolites. Effective absorptive performance has been observed for modified nanoclays [13,14].

Clay minerals as naturally occurring nanomaterials with around one nanometer thick silicate layers are inexpensive and environmentally friendly materials [15]. Most of them are composed of tetrahedral and octahedral sheets. These sheets are arranged in different ways, resulting in two categories of clay with either a 1:1 or a 2:1 layering. Sodium Montmorillonite (Na^+^-Mt) is a 2:1 clay mineral composed of a central octahedral Al sheet of Al_2_O_3_ composition between two tetrahedral SiO_2_ sheets [16,17]. The sheets are negatively charged due to isomorphic substitutions between cations. This charge is counter balanced by exchangeable cations located in the lattice structure, resulting in an increase in the hydrophilic character of clay minerals [18]. Montmorillonite (Mt) has a net negative surface charge, and can be used as an adsorbent for the removal of cationic pollutants [15,16]. Surface modification of Mt can improve both its properties and applications as an adsorbent of anionic organic pollutants in soil, water, and air. Several methods can be employed to modify Mt [19]. Ion exchange with organic ammonium cations is the most common method. Recently, researchers’ interests have been shifted towards the use of other organo-modifiers, such as quaternary phosphonium salts [20] and non-ionic surfactants [21]. Some researchers have modified clay minerals with (3-aminopropyl) triethoxysilane to form polymer nanocomposites, with interesting results [22]. According to the arrangement of polymer–clay nanocomposites, they are classified into three types; conventional composites, intercalated nanocomposites, and delaminated nanocomposites [19]. Polyionenes, or ionenes, are a class of polyelectrolytes where quaternary nitrogen atoms are located in the backbone [23], as opposed to in a pendent site [24]. Typical polyionenes can be obtained through the reaction of dihalides with a ditertiary amine [25]. The regular repetition of the charges on the backbone can be easily controlled through the changing of the reacting substrates. Simple and polymeric ionenes have been known to possess important biological activity [23]. Polyaspartates represent a type of poly amino acid with free carboxylic acid groups [26], which are biodegradable and super-absorbent, [27] as well as pH responsive polymer gels [28]. These polymers have been widely investigated in corrosion inhibition, [29] water treatment, biomineralization, [30] drug delivery, and tissue engineering [31].

We used a simply prepared nanocomposite material based on naturally available inorganic material such as Na^+^-Mt, modified with biodegradable antimicrobial-biodegradable polymers such as polyaspartate and the polyionene. Substituting the counter ion of the ionene gives the opportunity to control the properties of the resulting material in terms of its hydrophilic hydrophobic water properties and can thus control its ability to combine with various organic substrates [32,33].

In the current study, Na^+^-Mt was rendered organophilic in nature through modification with cationic polymers (polyionene) and with simple monomeric ionene compounds and was further modified by reaction with polyaspartate. The Mt-nanocomposites were investigated with respect to the adsorption of anionic dye such as Acid Blue 25. The effects of the chemical structure of ionenes on the adsorption capacity were studied. Moreover, kinetics, thermodynamic properties, and adsorption isotherms of the adsorption process were investigated.

## 2. Materials and Methods

### 2.1. Materials

Sodium-montmorillonite (Na^+^-Mt) (Cloisite^®^Na, Southern Clay Products, Gonzales, TX, USA) having cation exchange capacity (CEC) = 92.6 meq/100 g and the basal spacing = 1.16 nm) was received from Southern Clay Products, Gonzales, TX, USA (Table 1). Na^+^-Mt has a total oxide composition of 98.17% (60.12% of SiO_2_, 19.95% of Al_2_O_3_, 7.6% of Fe_2_O_3_, 4.63% of MgO, 3.43% of CaO, 2.44% of Na_2_O) [34]. Acid Blue 25 dye was purchased from Sigma-Aldrich (Steinheim, Germany) (Table 1). Dimethylformamide (DMF) was supplied by Acros Organics (New Jersey, NJ, USA). Poly(succinimide-co-aspartate) copolymer 1:1 residue ratio (aspartate: succinimide), *α,α’*-dichloro-*p*-xylene, benzyl chloride, methyl iodide, *N,N,N’,N’*-tetramethylethylenediamine (TMEDA), and *N,N*-dimethyldecylamine were obtained from Folia, Inc., (Birmingham, AL, USA).

### 2.2. Instrumentation

The characterization of the prepared samples was studied using routine analysis as described by Elsherbiny et al. [35,36] using the following instruments: FTIR absorption spectra (Bruker TENSOR-27 spectrophotometer ( Bruker, Billerica, MA, USA) in the wavenumber range of 400–4000 cm^−1^, and using KBr discs), Thermogravimetric analysis (TGA) under N_2_ (TA-Q500 thermographic analyzer, TA, Pittsburgh, PA, USA) in the temperature range of 30–800 °C with a scanning rate of 10 °C/min), Scanning electron microscopy (SEM) (JEOL JSM-6380 with an energy dispersive X-ray detector, JEOL, Akishima, Japan), Transmission electron microscopy (TEM) (JEOL 1011 CX, Tokyo, Japan), powder X-ray diffraction (PXRD) measurements were conducted using a Rigaku Ultima IV X-ray diffractometer ( JEOL, Akishima, Japan), and a Perkin Elmer Lambda 35 UV-vis spectrometer (Perkin Elmer, Waltham, MA, USA).

### 2.3. Synthesis of Ionene Compounds

Ionene samples were prepared using the method described by Rembaum et al. [25,37]. Synthesis details were given in the supporting information.

### 2.4. Chemical Modification of Na^+^-Mt with Ionene

Ionene clay nanocomposites (IC 1-3,) were prepared via reaction of Na^+^-Mt with the ionene compounds (I 1-3), respectively, using listed quantities in Table S1. The summarized procedure is: to aqueous suspension of clay, excess solution of ionene was added with continuous stirring at 40 °C for 48 h. The product was obtained by centrifugation at 5,000 rpm, washed with water, ethanol, and diethyl ether. The samples were dried under a vacuum at 40 °C overnight. Volhard’s method was used to determine the remaining halide ions [38] (as shown in Table S1). Elemental microanalysis is given in Table S2.

### 2.5. Preparation of Nanocomposites ICP-1-3

Nanocomposites ICP-1–3, were prepared by mixing poly(succinimide-co-aspartate) with ionene-clay organoclays (IC-1–3) using the quantities listed in Table S1. Typically, an aqueous solution of poly(succinimide-*co*-aspartate) was added to a suspension of organoclay in 30 mL H_2_O, and stirred overnight at 40 °C. The product was settled out by addition of a small quantity of ethanol. The resulting mixture was centrifuged at 5000 rpm, washed several times with water and dried under a vacuum. The proposed chemical structures of the resulting nanocomposites are included in Table S3.

### 2.6. Adsorption Studies

Adsorption experiments were carried out in batch equilibrium mode as mentioned by Elsherbiny et al. [35,36] More details were given in the supporting information.

## 3. Results

### 3.1. Mt-Composites

**Scheme 1:**
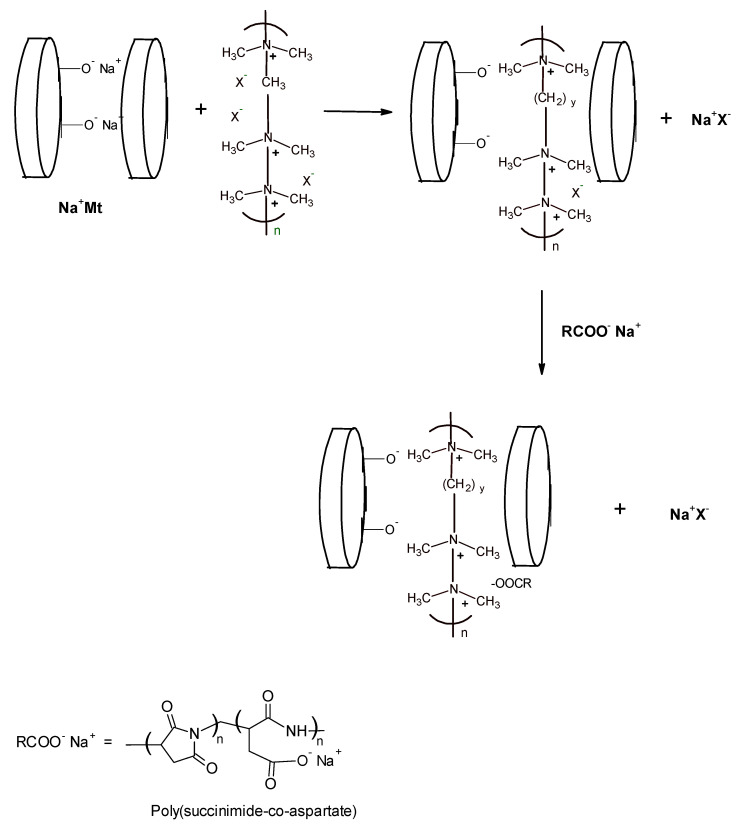
Synthetic route to ICP samples.

In the present work, polyionenes and a simple bis-quaternary ammonium salt were reacted in excess with Na^+^-Mt to provide an organophilic nature to the clay, and further modification with a polyaspartate afforded a large number of active sites that could bind with oppositely charged organic molecules (Scheme 1). The structure of the prepared ionenes and the ionene-clay compounds was confirmed by elemental microanalysis results, which are in good agreement with the calculated values (Table S2). For ionene-clay compounds, the precipitate was isolated by centrifugation and washed several times with H_2_O water, until AgNO_3_ tests indicated an absence of chloride in the filtrate. The remaining free chloride was determined by Volhard’s method (Table S2).

### 3.2. FTIR Spectra

**Figure 1:**
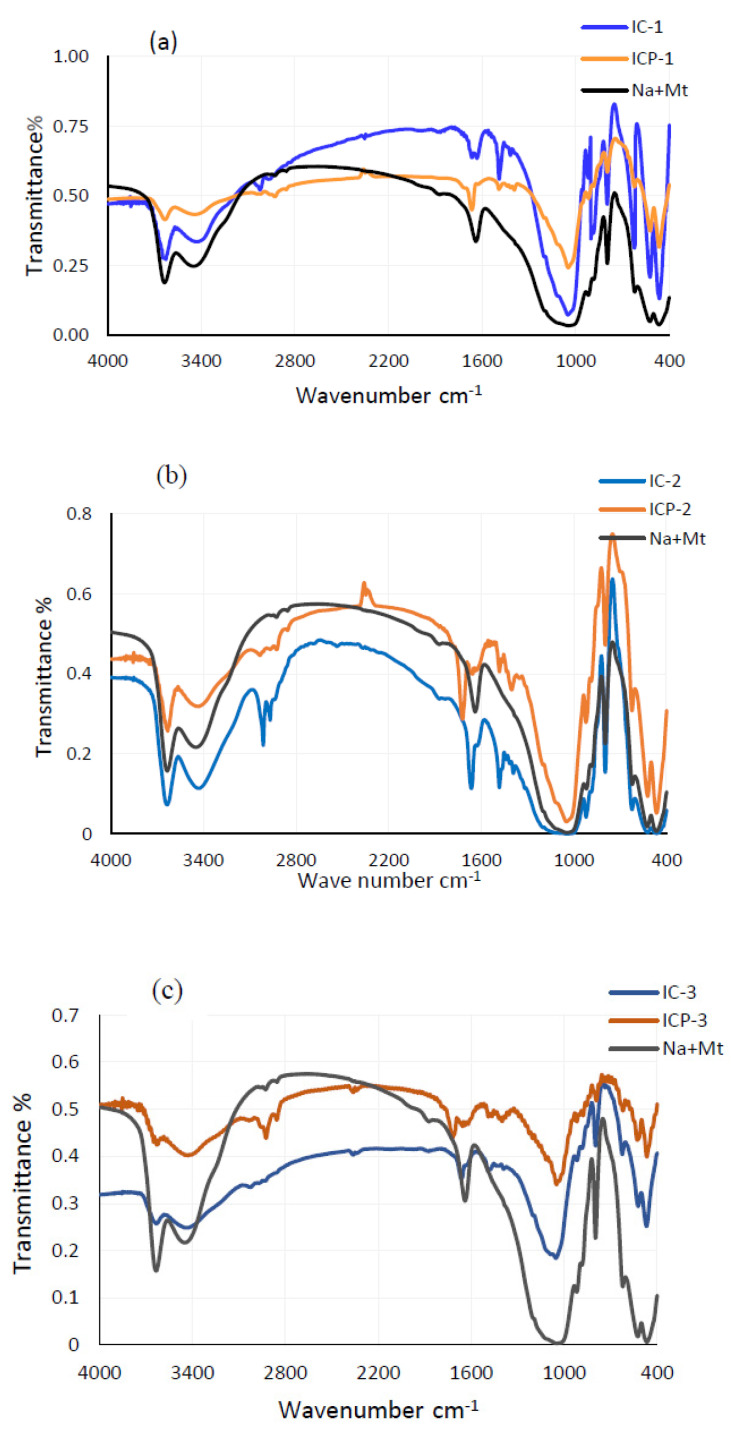
FT-IR Spectra of (**a**) IC-1, ICP-1, Na^+^-Mt; (**b**) IC-2, ICP-2, Na^+^-Mt; and (**c**) IC-3, ICP-3, Na^+^-Mt.

Functional groups on the surface and interlayers of the composites as well as the modification of Na^+^-Mt with ionenes and polyaspartate were identified using FTIR spectra as shown in Figure 1. Na^+^-Mt showed all the characteristic bands as mentioned by Elsherbiny et al. [35,36] The structures of the ionene-clays IC-1-3 were confirmed by FTIR spectra, which showed bands at 2944 cm^−1^, corresponding to the alkyl C–H stretch, and at 3022 cm^−1^, corresponding to the methylene C–H stretch, while the ammonium nitrogen appeared at 3446 and 1456 cm^−1^. The phenyl ring (1,4-substituted) band appeared at 1005 cm^−1^ (except for IC-4). A band at 958 cm^−1^ corresponds to N^+^C. The peaks at 3622 and 3436 cm^−1^ were due to clay OH stretching (Mg, Al–OH), and hydrogen-bonded water. The peak at 1049 cm^−1^ was due to Si–O–Si stretching. Modification of ionene-clay compounds with an aspartate polymer to yield the ICP nanocomposites was confirmed by the observation of additional peaks at 1716 and 1722 cm^−1^, which were attributed to the C=O and NH groups of the aspartate polymer, and a peak at 3406 cm^−1^ was attributed to the overlapping of NH and OH bands [39].

### 3.3. PXRD Analysis

The interaction of the polymer with the clay in the prepared nanocomposites were examined by PXRD. The XRD diffraction patterns of Na^+^-Mt (Figure S1) showed a characteristic peak at 2θ = 7.60°, which corresponds to a spacing of d = 11.64 Å. The modified ICP nanocomposites were shifted to lower theta values from 2θ = 7.60° to 2θ = 6.24, 6.04 and 5.16° for ICP-1, 2, and 3 respectively (Table S4). The distance shift ranged from 2.53 to 5.49 Å due to the cation-exchange between Na^+^ and the quaternary ammonium salt/polyaspartate. Since, ICP-1 has a simple ionone structure and ICP-2 and ICP-3 have polymeric structures, this results in a higher increase in the Mt interlayers.

### 3.4. SEM of ICP Samples

**Figure 2:**
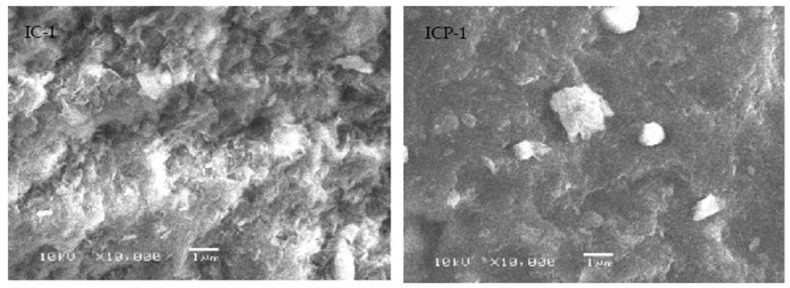

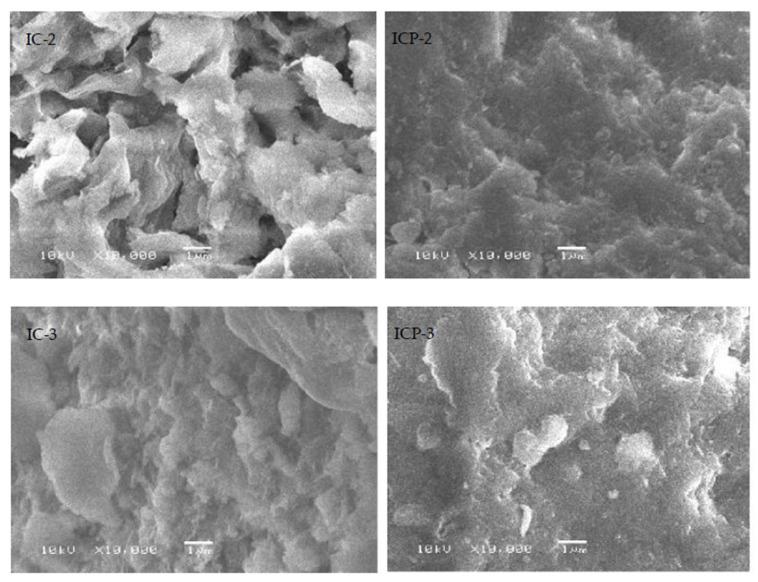
SEM of nanocomposite samples IC-1–3 and ICP-1–3.

The surface morphologies of the ICP-1–3 nanocomposites and their precursor IC-1–3 samples were examined using SEM (Figure 2). The surfaces of IC-1–3 samples appear rigid and rough, with sharp edges. Upon exchange of the chloride ions with polyaspartate ions, the surfaces became slightly smoother with less sharp edges and homogeneous surfaces with no agglomeration of the organic material or phase separation. Furthermore, the overall range of particle size domains was estimated at approximately <1 µm.

### 3.5. TEM of ICP Samples

**Figure 3:**
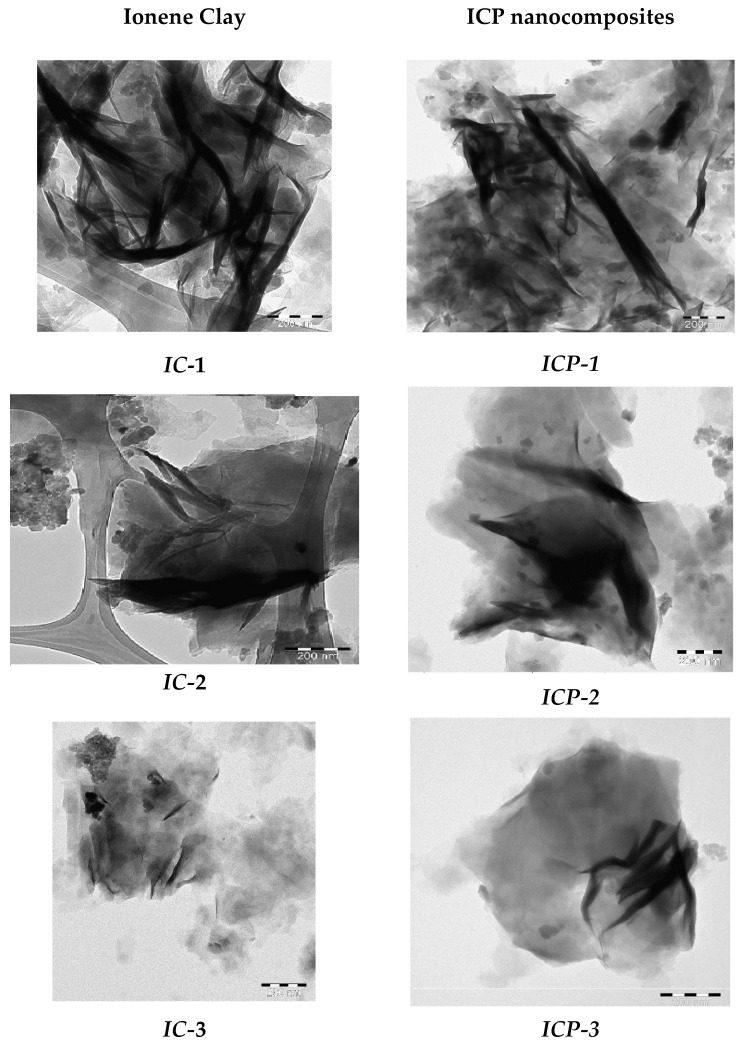
TEM of nanocomposite samples IC-1–3 and ICP-1–3.

As shown in Figure 3, TEM images offer further and qualitative assessment of structural characteristics of the nanocomposite. The dark and bold lines in the images of the ionene- Na^+^-Mt samples IC 1–3 represent the silicate layers where the nanocomposite structure contains primarily heavier elements than those present in both polyionene and ionene materials. Additionally, TEM images reveal a very good dispersion of the silicate layers with exfoliated particles while some maintained their original ordering. These observations support the results of XRD. After modification with polyaspartate, there is no significant change in the surface while maintaining very good dispersions and exfoliations.

### 3.6. TGA of Ionene Na^+^-Mt and Its Modified Derivatives

**Figure 4:**
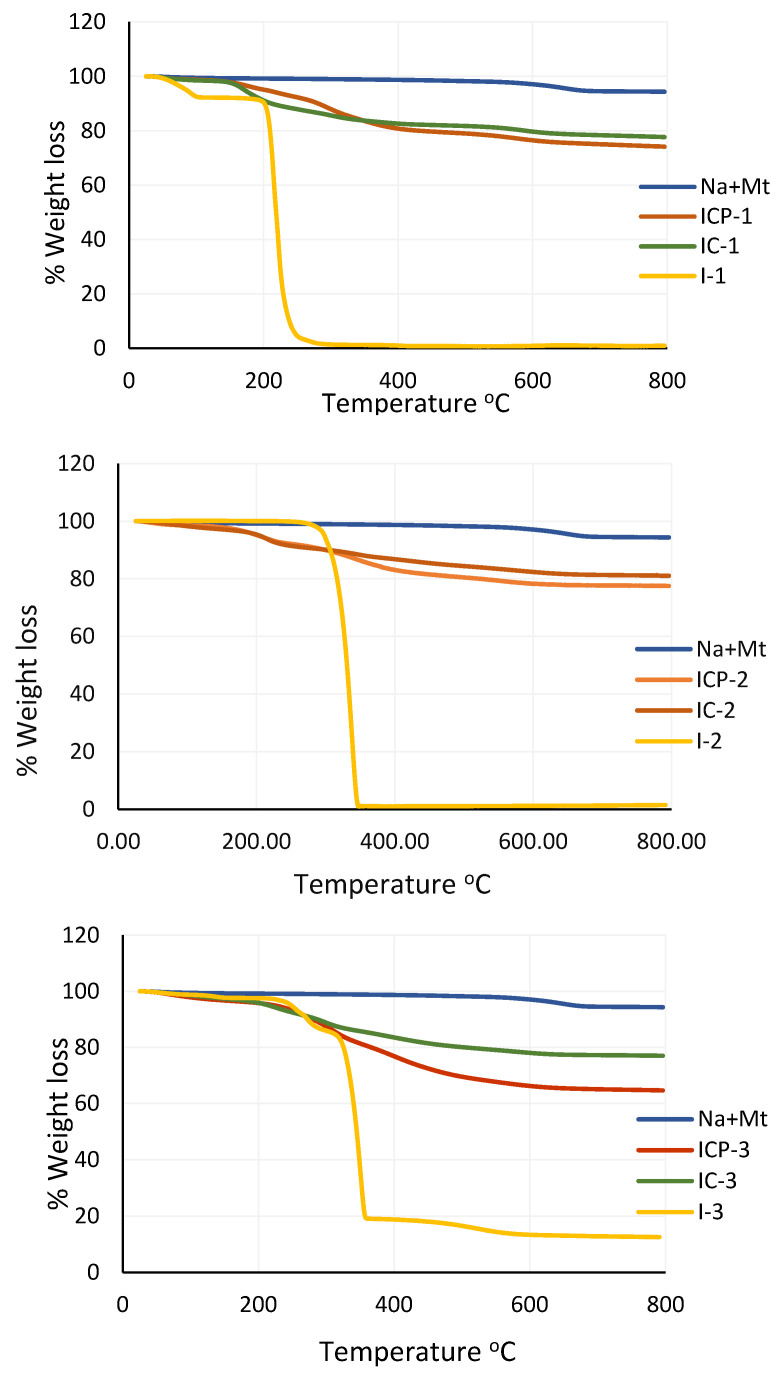
TGA of Na^+^-Mt, ionene and nanocomposite samples ICP-1–3.

The thermal stability of the nanocomposite (ICP-1), compared with those of Na^+^-Mt, ionene Na^+^-Mt (IC-1), and simple ionene (I-1) is presented in Figure 4. The intercalated nanocomposite sample ICP-1 and for IC-1 showed high thermal stability compared to simple ionene (I-1). TGA thermogram of I-1 showed two stages of weight loss; at approximately 105 °C, corresponds to a 10% weight loss due to the evaporation of moisture, and a major weight loss at 220 °C, was due to the degradation of the ammonium groups. When I-1 reacted with Na^+^-Mt to give ionene- Na^+^-Mt (IC-1), a major increase in thermal stability was observed, and the onset of degradation, at which approximately 6% weight loss occurs, is delayed to 171 °C. This was attributed to the evaporation of moisture and adsorbed water. A further two steps were observed at 306 and 589 °C due to the degradation of hydroxyl groups and ammonium groups, respectively. The nanocomposite ICP-1 showed similar behavior to IC-1, with three broad-range weight losses at 177, 296, and 578 °C, due to the evaporation of moisture and adsorbed water, and subsequent losses of structural hydroxyl groups and degradation of ammonium groups. The nanocomposite ICP-1 produced little residue compared to IC-1 due to the increased organic content over that of the Na^+^-Mt. Similar behavior was observed for the rest of materials, with ICP-2 and ICP-3 exhibiting decreasing amounts of residue. Sample IC-2 showed three stages of degradation at 79, 214, and 582 °C, while IC-3 showed three stages of degradation at 79, 214, and 592 °C. The composites ICP-2 and ICP-3 showed degradation steps at 47, 209, and 560 °C and at 65, 311, and 559 °C for ICP-2 and ICP-3, respectively. These degradation steps can be attributed to loss of moisture and adsorbed water, and to loss of structural hydroxyl groups and degradation of ammonium groups. The Na^+^-Mt samples are more stable than the ionene samples due to the greater modulus and the reduced thermal expansion coefficient of the clay compared to the ionene and aspartate polymer, which results in the inhibition of both oxygen diffusion and volatile products through the intercalated materials, and therefore leads to the obtained thermal stability for IC and ICP samples relative to the ionene samples.

### 3.7. BET Surface Area of ICP Nanocomposites

The BET surface areas (SBET) for the nanocomposites were calculated based on the N_2_ adsorption-desorption isotherms. The specific surface areas, mean pore diameters, and total pore volumes of Na^+^-Mt, ICP-1, ICP-2, and ICP-3 were determined and are listed in Table 2. Compared with the modified composites, Na^+^-Mt has the largest specific surface area. The compact packing of ionene in interlayers prevented the passage of N_2_, resulting in a decrease in SBET in the modified composites [40]. Thus, modification of Mt with polyaspartate was accompanied with an overall decrease in surface area and mean pore diameter (r). However, the total pore volume (VT) increased from ICP-1 to ICP-3 due to the intercalation of ionene in the interlayer space of Mt. This increment in the pore volume from ICP-1 to ICP-3 is in agreement with the shift in *2*θ to lower values in the X-ray pattern from ICP-1 to ICP-3. The specific surface areas of the three composites decreased in the order ICP-1 > ICP-2 > ICP-3, due to the chemical structure of the ionene linker. Ionene has a simple structure in the case of ICP-1. However, in the cases of ICP-2 and ICP-3, it has a more complex structure than ICP-1 which filled the pores and prevented the passage of N_2_ gas, resulting in a reduction of the surface area.

### 3.8. Adsorption Studies

#### 3.8.1. Effect of Ionene Structure on the Adsorption Efficiency

The dependence of the quantity adsorbed at equilibrium, *q_e_*, on the structure of the modified Mt, as well as on the initial concentration of AB25 is presented in Table 3. As shown in Table 3, *q_e_* was increased by increasing the initial concentration of AB25 for all three adsorbents, due to the high concentration gradient of AB25. Additionally, the quantity adsorbed on ICP-1 was the largest at all the examined initial concentrations, while *qe* of ICP-3 is higher than that of ICP-2. This phenomenon was attributed to two factors; the difference in the surface area of the adsorbents and their chemical structures [41]. Table 1 showed that the specific surface areas of the adsorbents increases in the order ICP-1 > ICP-2 > ICP-3, which agrees with the highest adsorption value of ICP-1. However, ICP-2 has a higher surface area than ICP-3, yet adsorbed a smaller quantity of AB25 than ICP-3. This result is related to the chemical structure of the composites. ICP-1 has two benzene rings, one on each side, which interact with the benzene rings in AB25 via π–π interactions [42]. Morevoer, the benzene ring has a negative inductive effect (-I). This effect leads to an increase in the positive charge on the nitrogen atom (positive center) of the composite which facilitates the electrostatic interaction with the anionic AB25. However, ICP-2 has an alkyl chain with (+I) attached to the nitrogen atom on both sides, leading to a reduction in the positive charge on the nitrogen atom, thus resulting in a smaller adsorbed amount of AB25 on the ICP-2 composite. ICP-3 has a benzene ring attached to its nitrogen atom on one side and the other side attached to an alkyl chain. Therefore, we expect that the π–π electron interaction between the benzene rings present in ICP-3 and the benzene rings in AB25 to enhance the attraction between ICP-3 and the dye molecules as well as the electrostatic interaction.

#### 3.8.2. Adsorption Kinetics

**Figure 5:**
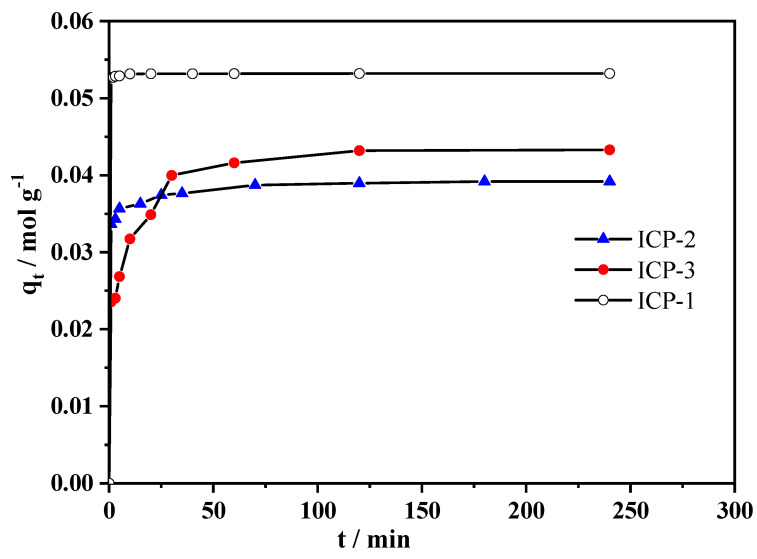
Pre-equilibrium adsorption of AB25 onto nanocomposites ICP 1-3 versus contact time at 25 °C, (ICP 1-3: 2.0 g/L), pH = 7, and [AB25] = 2 × 10^−4^ mol/L.

**Figure 6:**
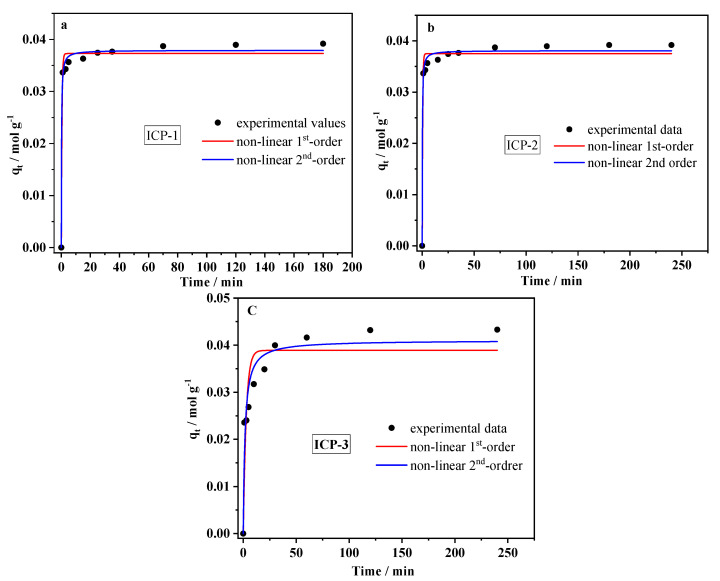
Non-linear kinetics model for the adsorption of AB25 on ICP nanocomposites. (**a**), (**b**) and (**c**) represent ICP 1–3 respectively.

The plot of the quantity of AB25 adsorbed onto the adsorbents, *q_t_*, as a function of the time, *t*, is shown in Figure 5. The initial adsorption is very rapid in the case of ICP-1 and is rapid for ICP-2 and ICP-3, due to the adsorption of AB25 onto the external surface of the particles. Subsequently, the process slowed down and reached equilibrium within a few minutes, which was attributed to the slower diffusion of the dye molecules into the pores of the adsorbents, since many of the available external sites had been occupied during the initial stage [41]. Additionally, from Figure 5, it can be observed that the adsorbents have different adsorption equilibration times (10 min for ICP-1, 70 min for ICP-2 and 120 min in the case of ICP-3). This was attributed to the difference in their specific surface areas (Table 2) [41], and correlates well with data in Table 3. Furthermore, two kinetic models were used to analyze the experimental data; the non-linear and linear forms of pseudo-first, and pseudo-second order models. Complete descriptions of the two models have been provided in the supporting information. The kinetic parameters, along with the correlation coefficients (*R^2^*), which were calculated from the non-linear (Figure 6) and linear plots (Figures S2 and S3) of each model, are listed in Table 4. The correlation coefficient (*R^2^)* of the linear and non-linear pseudo-second order model is higher than that of the linear and non-linear pseudo-first order model and very close to unity (linear form) for all adsorbents. This indicates that the kinetic data for all adsorbents were best represented by the pseudo-second order model. Moreover, the adsorption capacities, which were calculated from the pseudo-first order mode (*q_e,cal_*), were significantly different from the experimental values (*q_e,exp_*), whereas the values of *q_e_,_cal_* of the pseudo-second order model were very close to those of *q_e,exp_*. Therefore, it would have been reasonable to conclude that the pseudo-second order model fit the adsorption of AB25 onto the three adsorbents well. The intraparticle diffusion model of Weber was applied to classify the steps which were happened during the adsorption process. Detailed information about this model is provided in the supporting information. As shown in Figure S3, the plots of *q_t_* versus *t^½^* were linear with two intersected regions and did not pass through the origin for the three composites. This indicates that intraparticle diffusion plays a major role in the kinetics of the system; however, it is not the rate limiting-step [35]. The two intersected regions, which are presented in Figure S5; confirm that the adsorption occurred in two stages: a bulk diffusion of the AB25 onto the external surface of the adsorbents, followed by intraparticle diffusion of the dye into the pores [36]. Hence, the adsorption of AB25 onto ICP-1, ICP-2, and ICP-3 is defined by the pseudo-second order model combined with intraparticle diffusion.

#### 3.8.3. pH Effect

The initial pH of the dye solution is an important factor in controlling the adsorption process, particularly the adsorption capacity. The initial pH of the dye solution was varied from 3 to 11, and the results are presented in Figure S5. The equilibrium adsorption capacity, *q_e_,* of AB25 onto ICP-1, ICP-2, and ICP-3 composites decreased slightly as the initial pH of the dye solution increased from 3 to 11. The adsorption mechanism can be elucidated by the electrostatic interactions between the negatively charged dye ions and the positively charged sites (N atom) of the three composites, in addition to the π–π electron interactions between the benzene rings of the dye molecule and the benzene rings of ICP-1 and ICP-3. AB25 is an anionic dye containing one sulfonic group (–SO_3_^−^Na), which dissociates in water to form a sodium cation (Na^+^) and a sulfonate anion (–SO_3_^−^). At acidic pH, the sulfonic group can be protonated to the neutral form (–SO_3_H). However, in higher acidic solutions, the sulfonic group retains a negative charge as it has a *pK_a_* value lower than zero [43]. At basic pH, the hydroxyl group competes with AB25, which results in a decrease in the adsorption capacity [35,44].

#### 3.8.4. Adsorption Isotherms

The adsorption isotherm supplies information about the distribution of the adsorbate molecules between the liquid phase and the solid phase, at the equilibrium state. To investigate the adsorption mechanism, three adsorption isotherm models were examined based on the adsorption equilibrium data. These models are the Freundlich, Langmuir, and Dubinin– Radushkevich (D–R) models [44], which are described in the supporting information. The estimated model parameters are listed in Table 5. As can be seen in this Table, the adsorption equilibrium data for the adsorbents were best represented by Freundlich isotherm, according to the values of *R^2^*. The better simulation of experimental data by Freundlich model was attributed to the heterogeneity of the adsorbent surfaces and the formation of multilayers of adsorbed AB25 on the surfaces [45]. The reciprocal of the intensity factor (*1*/*n*) was less than unity in the case of ICP-3, and increased with increasing temperature, which implies that the adsorption of AB25 onto ICP-3 is favorable [36]. In contrast, the 1/*n* value is higher than unity for ICP-1 and ICP-2. Moreover, the increase in the Freundlich constant, *K_F_*, with increasing temperature in the cases of ICP-1 and ICP-3 indicates that the adsorption process is favorable at high temperature and is endothermic in nature [46]. Conversely, the adsorption capacity of ICP-2 decreased with an increase in temperature, implying that the process is exothermic. The Langmuir model provided lower quality fits for the adsorption data of ICP-1 and ICP-2; however, it could not reasonably represent the experimental data of ICP-3. By using the D-R model, the value of the mean adsorption energy (*E*) was calculated, which provides evidence of the adsorption mechanism. The E value for the adsorption of AB25 onto ICP-2 was 6.23 kJ·mol^−1^ at 298 K, implying that the adsorption is a physical process. However, in the case of ICP-1, the E value was 11.13 kJ·mol^−1^ at 298 K, indicating that the adsorption is governed by an ion-exchange mechanism [8]. This model did not fit the adsorption data of AB25 on the ICP-3 surface.

#### 3.8.5. Thermodynamic Parameters

The thermodynamic studies provided important information on energy changes in the adsorption processes. The thermodynamic parameters, including the standard free energy change (Δ*G*°), standard enthalpy change (Δ*H*°), and standard entropy change (Δ*S*°), were calculated from the experimental data at three different temperatures using relative equations, as described in the Supporting Information. The estimated values are listed in Table 6. The distribution coefficient, *K_d_*, of the adsorption of AB25 onto ICP-1 and ICP-3 increased with an increase in temperature from 298 to 318 K, while *K_d_* decreased with an increase in temperature in the case of ICP-2. The positive values of ΔH° in the case of ICP-1 and ICP-3 indicate that the adsorption was endothermic, which is in good agreement with the values of *K_d_.* Moreover, the small positive value of Δ*H−1* (<40 kJ·mol^−1^) of ICP-1 indicates that the adsorption process was controlled by physical adsorption [47]. In contrast, the large positive Δ*H*° (>40 kJmol^−1^) value of ICP-3 suggests that the process was dominated by chemical adsorption [44]. The adsorption of AB25 onto ICP-2 had a negative Δ*H*° value, which confirmed that the process is exothermic in nature. This is due to the higher *K_d_* observed at 298 K, in comparison with 318 K. The process was found to be spontaneous for all three composites, which was indicated by negative ΔG° values. Maximum values of ΔG^0^ were observed at 318 K. Additionally, the AB25–ICP-1 system has the most negative value of ΔG°, which corresponds to the most spontaneous system and reflects the high ability of ICP-1 to adsorb AB25. The positive ΔS° value for all three adsorbents revealed that the disorder increased during the adsorption process [48].

#### 3.8.6. Mechanism of Adsorption

**Figure 7:**
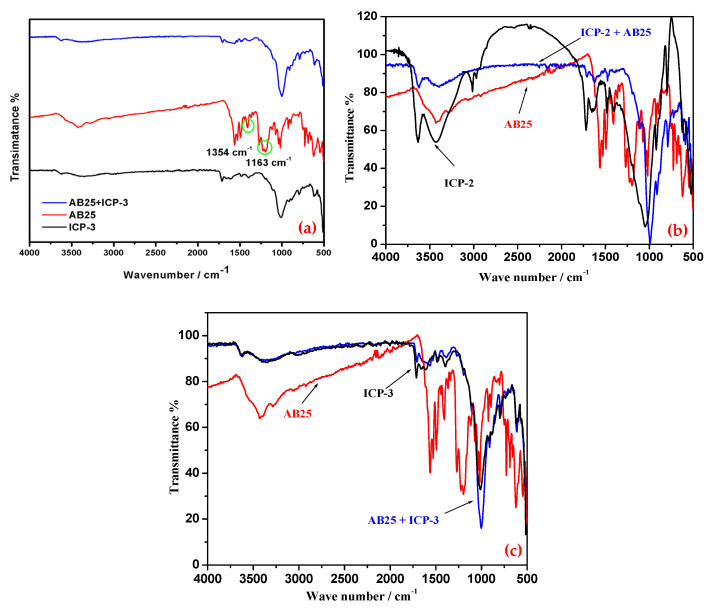
FTIR spectral comparison of adsorbed AB25 onto ICP nanocomposites with free AB25 and ICP samples (**a**): ICP-1; (**b**): ICP-2; (**c**): ICP-3).

FT-IR spectra for AB25, after adsorption onto the three nanocomposites, were recorded to clarify the mechanism of adsorption and are depicted in Figure 7a–c. The peak at 958 cm^−1^, corresponding to N^+^C in the ICP nanocomposites, and two bands at 1354 cm^−1^ and 1163 cm^−1^, which correspond to the S=O stretching in AB 25, are completely absent after adsorption. This suggests the electrostatic attraction between the –SO_3_^−^ group (negative dipole) of the Acid Blue 25 dye molecule and −N^+^C (positive dipole) in ICP-1, ICP-2, and ICP-3. This indicates that the adsorption process took place via an electrostatic interaction. Additionally, the observed peaks at 1716 and 1722 cm^−1^, which were attributed to the C=O and NH groups of the aspartate polymer, decreased in intensity after adsorption.

#### 3.8.7. Comparison with Other Adsorbents

In order to evaluate the usefulness of ICP-1–3 nanocomposites as adsorbents for AB25, their maximum adsorption capacity, *q_max_* (from Langmuir isotherm) with those of absorbents previously reported in the literature were compared [49,50,51,52,53] (Table 7). The composites are good candidates for the removal of AB25 from an aqueous solution compared with the other adsorbents. Moreover, they are effective at ambient conditions (neutral medium, 25 °C) and the composites were synthesized from low cost and eco-friendly materials.

## 4. Conclusions

Surface-modified Na^+^-Mt using different ionene compounds as a linker between polyaspartate and Na^+^-Mt are able to remove the anionic dye, Acid Blue 25, very effectively. Both the surface area and the chemical structure of ionene play an important role in the adsorption efficiency of the prepared composites. From the study of the adsorption mechanism by FTIR, we can conclude that the adsorption process is governed by electrostatic interactions between cationic active sites on the adsorbent surface and anionic AB25, in addition to the π–π electron interaction between the benzene rings of the adsorbate and absorbent. The adsorption process was spontaneous for all adsorbents, as indicated by negative values of Δ*G^o^*. The small positive value of Δ*H*° (<40 kJ mol^−1^) for ICP-1 indicates that the adsorption process was governed by physical adsorption. The large positive Δ*H*° (>40 kJ mol^−1^) value of ICP-3 suggests that the process was dominated by chemical adsorption. The adsorption of AB25 onto ICP-2 has a negative Δ*H°* value, which confirmed that the process is exothermic in nature.

## Figures and Tables

**Table 1:** The characteristics of acid blue 25 dye.

Dye	Chemical Structure	Molar Mass (g/mol)	Dye Purity	Molecular Formula	$\lambda_{max}$ (nm)	CI
Acid Blue 25	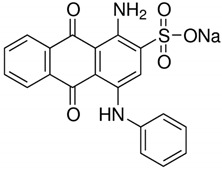	416.38	Pure	C_20_H_13_N_2_NaO_5_S	600	62055

**Table 2:** Specific surface area and pore radius of the ICP nanocomposites.

Material	Specific Surface Area (m^2^ g^−1^)	Aver. Pore Diam., r (nm)	Total Pore Volume, VT (mL g^−1^)
Na^+^-Mt	725.00	1.8	
ICP-1	150.50	1.32	0.065
ICP-2	120.19	1.32	0.522
ICP-3	100.20	1.15	0.652

**Table 3:** The dependence of the equilibrium adsorbed amount (*q_e_*) on the initial concentration of AB25 (*C_0_*) and the structure of the adsorbent at 25 °C.

Surface	*C_0_*/10^−4^ (mol L^−1^)	*q_e_*/10^−2^ (mol g^−1^)
ICP-1	0.70	2.47
	1.00	3.71
	1.75	4.33
	2.00	5.32
	2.50	6.17
ICP-2	0.70	1.09
	1.00	1.64
	1.75	3.12
	2.00	4.33
	2.50	4.41
ICP-3	0.70	1.45
	1.00	1.95
	1.75	3.65
	2.00	3.92
	2.50	5.10

**Table 4:** Kinetics data with their correlation coefficient (R^2^) for the adsorption of [AB25] = 2.0 × 10^−4^ mol/L over the nanocomposites at 25 °C.

Adsorbents	Pseudo-First Order			Pseudo-Second Order				Intra-Particle Diffusion			
	*q_e_* (10^−4^ mol g^−1^)	*k_1_* (min^−1^)	*R^2^*	*q_e_,_exp_* (mol g^−1^)	*q_e_,_cal _* (mol g^−1^)	*k_2 _* (10^3^ mol g^−1^ min^−1^)	*R^2^*	*k_p1 _* (mol g^−1^ min^−0.5^)	*R^2^*	*k_p_*_2_ (mol g^−1^ min^−0.5^)	*R^2^*
Linear form											
ICP-1	8.97	0.102	0.871	0.053	0.050	0.126	1.00	0.040	0.959	3.42	0.745
ICP-2	94.6	0.051	0.938	0.039	0.039	13.900	0.999	0.016	0.876	29.60	0.941
ICP-3	218	0.445	0.445	0.043	0.044	35.400	0.999	0.008	0.953	61.60	0.795
Non-linear form											
ICP-1	373	2.300	0.983	0.053	0.038	0.162	0.992	-	-	-	-
ICP-2	375	2.500	0.981	0.039	0.038	0.153	0.991	-	-	-	-
ICP-3	389	0.363	0.828	0.043	0.410	0.015	0.921	-	-	-	-

**Table 5:** Isotherm parameters for the adsorption of AB25 dye over the nanocomposites.

Model	Parameters	Temperature (K)								
		298			308			318		
		ICP-1	ICP-2	ICP-3	ICP-1	ICP-2	ICP-3	ICP-1	ICP-2	ICP-3
Langmuir	*q_max_* (mol/g)	0.129	0.043	-	0.189	0.051		0.182	0.045	-
	*K_L_* × 10^4^ (L/mol)	27.90	0.787	-	19.3	0.708		22.00	0.595	-
	*R^2^*	0.895	0.948	-	0.782	0.697		0.887	0.833	-
Freundlich	*1/n*	1.630	1.420	0.921	1.42	1.470	0.954	1.350	1.530	0.998
	*K_F_* (mol/g (L/mol)^1/*n*^)	0.595	423.00	1.860	2.30	152.0	10.670	3.620	111	22.700
	*R^2^*	0.930	0.995	0.956	0.95	0.960	0.967	0.984	0.976	0.970
D-R	*q_m_* (mol/g)	2.790	48.500	211.00	8.870	28.190	-	6.600	22.700	-
	*K_D-R_* (mol^2^·J^−2^)	−0.004	−0.013	−124.00	−0.010	−0.011	-	−0.004	−0.011	-
	*E* (kJ·mol^−1^)	11.130	6.230	0.064	10.530	6.700	-	10.980	2.160	-
	*R^2^*	0.931	0.995	0.955	0.952	0.952	-	0.976	0.974	-

**Table 6:** Thermodynamic parameters of adsorbed AB25 dye over the nanocomposites. [ICP-1-3] = (2 g/L) and [AB25] = 2.0 × 10^−4^ mol/L.

Adsorbent	Temp (K)	*K_d_* × 10^3^ (g·L^−1^)	Δ*H* (kJ·moL^—1^)	Δ*G* (kJ·moL^−1^)	Δ*S* (J·moL^−1^·K^−1^)
ICP-1	298	25.060	8.181	−25.15	111.8
	308	29.750		−26.27	
	318	30.810		−27.38	
ICP-2	298	0.582	−11.5	−15.88	14.69
	308	0.569		−16.02	
	318	0.434		−16.17	
ICP-3	298	0.250	127.2	−14.6	475.9
	308	4.119		−19.36	
	318	6.163		−24.12	

**Table 7:** Comparison of maximum adsorption capacity of the adsorption of AB 25 onto various adsorbents reported in the literature.

Adsorbents	*q_max_* (mg/g)	Conditions	Reference
Waste tea activated carbon	203.3	300 K, m = 0.2 g, V = 0.25 L	[49]
Phosphoric acid treated rubber leaf powder	28.09	320 K, pH 2, m = 0.1 g	[50]
Treated *Shorea dasyphylla* Sawdust	24.39	300 K, pH 2, m = 0.1 g, v = 0.05 L	[51]
Natural Sepiolite	53.78	293 K, pH 3, V = 0.25 L	[52]
Zeolite	64.2	300 K	[53]
Zeolite-CTAB	112.4	333 K	[53]
ICP-1	53.7	298 K, pH 7, m = 0.04 g, V = 0.006 L	This study
ICP-2	17.9	298 K, pH 7, m = 0.04 g, V = 0.006 L	This study

## Supplementary Materials

The following are available online at https://www.mdpi.com/2073-4360/12/12/2843/s1, Figure S1. PXRD of Na^+^-Mt and nanocomposite samples ICP 1-3, Figure S2. Linear plot of Pseudo-first-order model of AB25 adsorbed over nanocomposites ICP 1-3 at 25 °C, (ICP 1-3: 2.0 g/L), pH = 7, and [AB25] = 2 × 10^−4^ mol/L, Figure S3. Linear plot of Pseudo-second-order model of AB25 adsorbed over nanocomposites ICP 1-3 at 25 °C, (ICP 1-3: 2.0 g/L), pH = 7, and [AB25] = 2 × 10^−4^ mol/L, Figure S4: Intraparticle diffusion plot for adsorption of AB25 over nanocomposites ICP 1-3 at 25 °C, (ICP 1-3: 2.0 g/L), pH = 7, and [AB25] = 2 × 10^−4^ mol/L., Figure S5: Effect of pH on the equilibrium adsorption of AB25 over nanocomposites ICP 1-3 at 25 °C, (ICP 1-3: 2.0 g/L), and [AB25] = 2 × 10^−4^ mol/L., Table S1. Summary of the amounts used for preparation of ionene-clay (IC-3-5) and nanocomposites (ICP-3-5) and their yield, Table S2. Elemental microanalysis of the prepared ionene and ionene-clay compounds, Table S3: Proposed Chemical Structure of ICP Samples, Table S4. Interlayer d-spacing and spacing shift.
