# Supplementary material for: Polyaspartate-Ionene/Na+-Montmorillonite Nanocomposites as Novel Adsorbent for Anionic Dye; Effect of Ionene Structure
Source: Polymers (Basel). 2020 Nov 29;12(12):2843. doi: 10.3390/polym12122843 (PMC7759794; doi:10.3390/polym12122843)
Supplement: Supplementary file 1 [file polymers-12-02843-s001.pdf]

polymers

MDPI

Article

Polyaspartate-Ionene/Na$^+$-Montmorillonite Nanocomposites as Novel Adsorbent for Anionic Dye; Effect of Ionene Structure

Hany El-Hamshary [1,3*], Abeer S. Elsherbiny [2,3*], Mohamed H. El-Newehy[1,3,*] and Mohamed E. EL-Hefnawy[2,3]

1 Department of Chemistry, College of Science, King Saud University, Riyadh 11451, Saudi Arabia;
2 Department of Chemistry, Rabigh College of Arts and Sciences, King Abdulaziz University, Jeddah 21911, Saudi Arabia
3 Department of Chemistry, Faculty of Science, Tanta University, Tanta 31527, Egypt;

Correspondence: melnewehy@ksu.edu.sa; Tel.: +96-6467-5894 (M.H.E.); abeer.elsherbiny@tanta.science.edu.eg (A.S.E.); helhamshary@ksu.edu.sa (H.E.)

Received: 22/10/2020; Accepted: date; Published: date

Synthesis of ionene compounds

Synthesis of poly(dimethylene, xylylene ionene) (PDMXI) (I-1): Ionene samples were prepared according to the method described by Rembaum *et al.* .The synthesis of I-1 is typical: a solution of α,α'-dichloro-*p*-xylene (6.13 g, 35 mmol) in DMF (20 mL) was mixed with TMEDA (4.07 g, 35 mmol) and heated to 40 °C, under nitrogen, for 5 days, resulting in 8.96 g of I-1(87%). FT-IR (KBr, cm-1): 3016(alkyl C–H), 3020 (methylene C–H stretch), 3411 and 1480 (ammonium nitrogen). ^{1}H NMR (400 MHz, DMSO-d_6): δ(ppm) 3.1 (t, 12H, CH_3), 4.16 (4 H, CH_2), 4.65 (4H, CH_2), 7.68 (4H, phenyl).

Synthesis of poly-2,2-ionene (PI) (I-2): Poly-2,2-ionene (PI) was prepared following the procedure for (PDMXI), using the following quantities: 1,2-dichloroethane (4.02 g, 40 mmol), TMEDA (4.65 g, 40 mmol), in DMF (20 mL) to yield 5.7 g I-2 (65%). FT-IR (KBr, cm^{-1}): 3009 (alkyl C–H stretch), 2969 (methylene C–H stretch), 3480, 1480, and 1404 (ammonium nitrogen). ^{1}H NMR (400 MHz, DMSO-d_6): δ(ppm) 3.3 (t, 12H, CH_3), 3.87 (4 H, CH_2), 4.65 (4H, CH_2).

Synthesis of I-3: A solution of benzyl chloride (9.11 g, 72 mmol) in DMF (30 mL) was mixed with TMEDA (4.063 g, 35 mmol) and heated to 40 °C, under nitrogen, for 48 h to yield 8.3 g I-3 (63%). FTIR (KBr, cm^{-1}): 2920 (alkyl C–H stretch), 3020 (methylene C–H stretch), 3475, 3392, and 1487 (ammonium nitrogen). ^{1}H NMR (400 MHz, *DMSO-d_6*): δ(ppm) 3.30 (*s*, 12H, CH_3), 3.68 (d, 4 H, CH_2,), 4.5 (s, 4H, CH_2), 7.6 (m, 10H, benzene).

Table S1. Summary of the amounts used for preparation of ionene-clay (IC-3-5) and nanocomposites (ICP-3-5) and their yield.

Sample code	Ionene			Clay	mole ratio of clay-	Yield
	code	g	mmol X^-/g	(g)	ionene	(g)
IC-1	I-1	0.898				2.01
IC-2	I-2	0.810	3.7	2	1:2	2.03
IC-3	I-3	1.110				1.84

Sample code	Ionene -clay			poly(succinimide-*co*-aspartate)		Yield
	code	g	mmol X^-/g	g	mmol	(g)
ICP-1	IC-1		1.96			2.20
ICP-2	IC-2	1.5	1.52	1.42	10	1.11
ICP-3	IC-3		3.03			1.39

Table S2. Summary of the amounts used for preparation of ionene-clay (IC-3-5) and nanocomposites (ICP-3-5) and their yield.

Inonene	%C		%H		%N		ammol X-/g
Code	Calc.	Found	Calc.	Found	Calc.	Found	
I-1	57.73	57.73	8.25	8.96	9.62	8.14	3.31
I-2	44.65	44.60	9.30	9.82	13.02	13.09	4.52
I-3	65.03	58.79	8.19	8.69	7.58	7.10	2.19
IC-1	-	14.20	-	2.36	-	2.78	1.13
IC-2	-	7.18	-	2.21	-	3.52	1.01
IC-3	-	15.84	-	3.15	-	3.52	2.02

[a] Determined by Volhard's titration method.

Table S3. Proposed chemical structure of nanocomposites (ICP-3-5).

Sample	Proposed structure
ICP-1	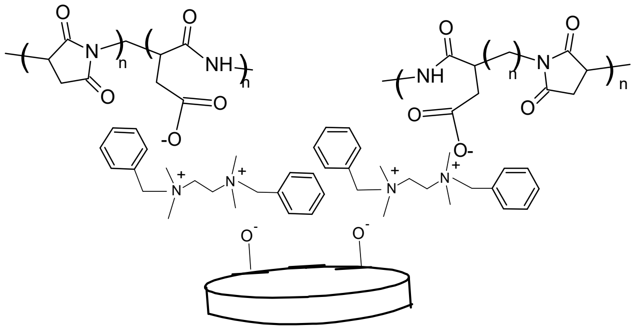
ICP-2	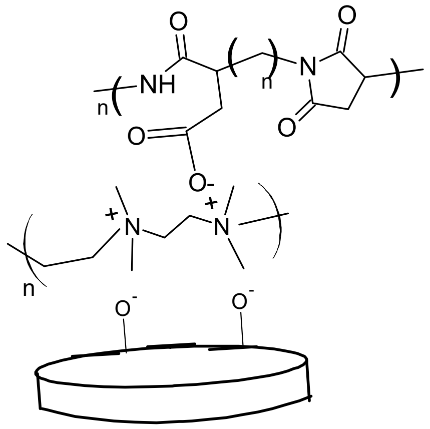
ICP-3	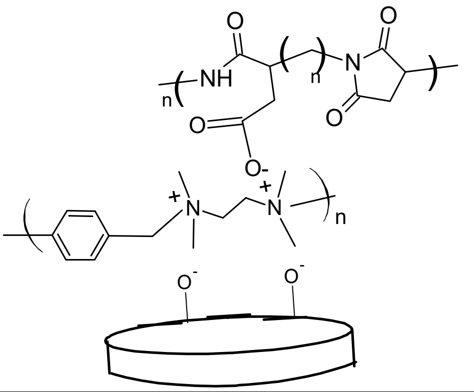

Adsorption studies

Adsorption experiments were carried out in batch equilibrium mode. Typically, 3 mL of Acid Blue 25 (AB25) with a concentration of 4 x 10-4 molL^{-1}, at pH 7, was added to 3 mL of adsorbent (ICP-1, ICP-2 or ICP-3) suspension (4 g/L) in a 100 mL capped Erlenmeyer flask. The flasks were shaken in a thermostatic water shaker bath (Unitronic -Selecta) at 60 rpm and 25 ± 0.2 °C to reach equilibrium. After equilibrium was attained and verified, samples were separated by centrifugation at 8000 rpm for 10 min. (Centrifuge-BL II Selecta). The concentration of the non-adsorbed dye in solution was determined from the measured absorbance at its λ_{max} = 604 nm using a UV-vis spectrometer. The equilibrium adsorption, q_e, was calculated using Equation 1:

$$q_e = (C_o - C_e)V / m \quad (1)$$

where C_o and C_e are the initial and equilibrium concentrations (mol L^{-1}) of AB25, respectively, V is the volume (mL) of solution, and m is the mass of the used adsorbent used in (g). The effect of pH on the adsorption of dye was studied in the range of pH 3–11. Dye solutions were prepared with distilled water and the pH was controlled with phosphate buffer. The experiments were conducted

at three different temperatures; 25, 35, and 45 ± 0.2 °C. The procedures for the kinetic experiments were identical to those of the equilibrium tests. At predetermined moments, dye solution was centrifuged and the concentration of the non-adsorbed AB25 was determined spectrophotometrically. The adsorbed quantity of AB25 at time *t*, q_t, and the removal percentage of AB25, % R) were calculated according to Equations 2 and 3, respectively:

$$q_t = (C_o - C_t)\ V / m \quad (2)$$

$$\%\ \mathrm{R} = ((C_o - C_t) / C_o)\ x\ 100 \quad (3)$$

where C_t (molL^{-1}) is the non-adsorbed dye concentration at time t. All experiments were done in duplicate and the average values were reported.

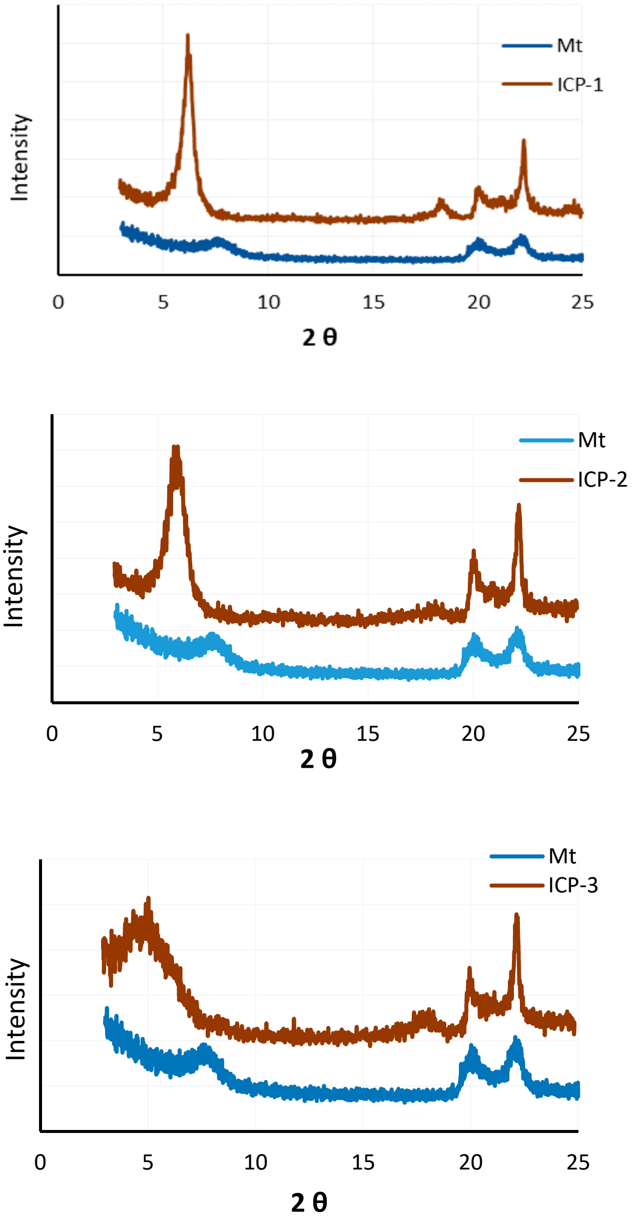

Figure S1. PXRD of Na^{+}-Mt and nanocomposite samples ICP 1-3.

Table S4. Interlayer d-spacing and spacing shift.

Material	2θ	d spacing / Å	Layer expansion / Å
Na^+-Mt	7.600	11.64	
ICP-1	6.240	14.17	2.53
ICP-2	6.040	14.63	2.99
ICP-3	5.160	17.13	5.49

Kinetic models

To inspect the mechanism of adsorption, two kinetic models were used to analyze the adsorption data; namely, non-linear and linear forms of pseudo-first-order (Eqs. 4, 5, respectively) and non-linear and linear forms pseudo-second order (Eqs. 6, 7, respectively) (1).

$$q_t = q_e(1\text{-}exp\text{-}k_1 t) \tag{4}$$

$$Log\ (q_e - q_t) = log\ q_e - (k_1/2.303)\ t \tag{5}$$

where k_1 is the adsorption rate constant. The values of k_1 and q_e were evaluated from the slope and intercept of the relation between log ($q_e - q_t$) and t, respectively.

$$q_t = k_2\ q_e^2 t/(1+q_e k_2\ t) \tag{6}$$

$$t\ /q_t = 1/k_2 q_e^2 + t/q_e \tag{7}$$

where k_2 is the second-order rate constant of adsorption.

Moreover, the kinetic data were introduced into intraparticle diffusion model which is expressed by equation (8) (2):

$$q_t = k_p\ t^{1/2} + C \tag{8}$$

k_p is the rate constant of the intraparticle diffusion (mol /g $min^{0.5}$) and C is a constant (mol /g) presenting data about the boundary thickness of the layer. The bigger C-value, the more influence of the boundary layer.

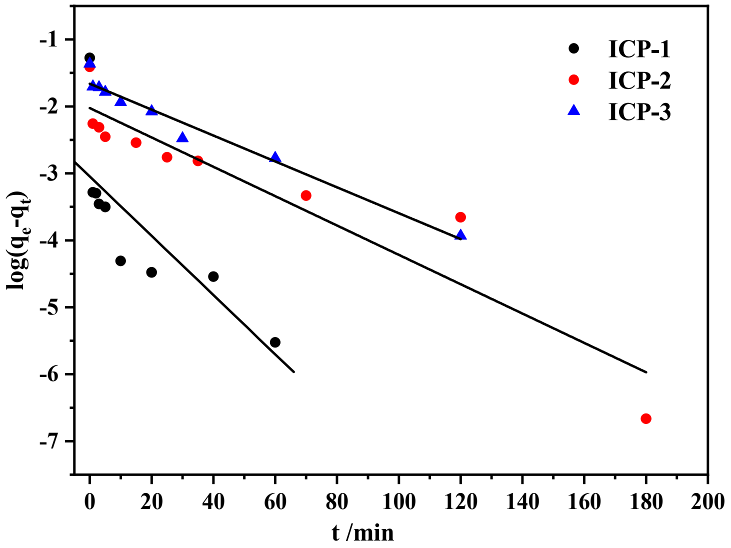

Figure S2. Linear plot of Pseudo-first-order model of AB25 adsorbed over nanocomposites ICP 1-3 at 25 ºC, (ICP 1-3: 2.0 g/L), pH = 7, and [AB25] = 2 x 10^{-4} mol/L.

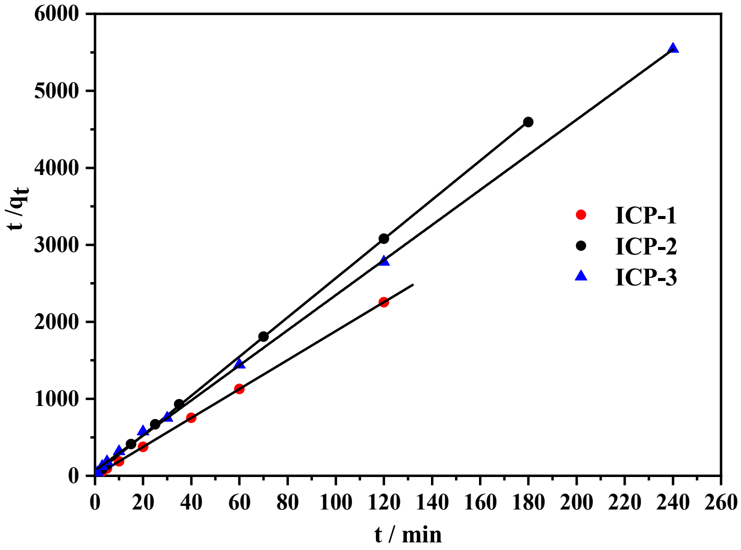

Figure S3. Linear plot of Pseudo-second-order model of AB25 adsorbed over nanocomposites ICP 1-3 at 25 ºC, (ICP 1-3: 2.0 g/L), pH = 7, and [AB25] = 2 x 10^{-4} mol/L.

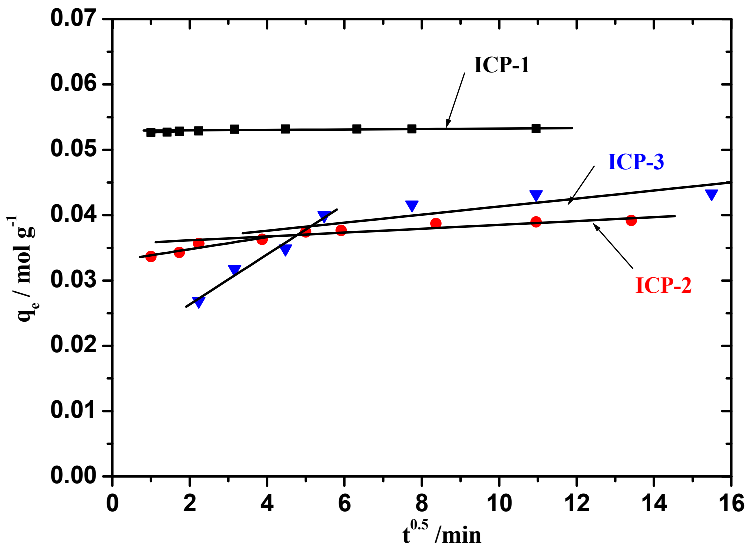

Figure S4. Intraparticle diffusion plot for adsorption of AB25 over nanocomposites ICP 1-3 at 25 °C, (ICP 1-3: 2.0 g/L), pH = 7, and [AB25] = 2 x 10^{-4} mol/L.

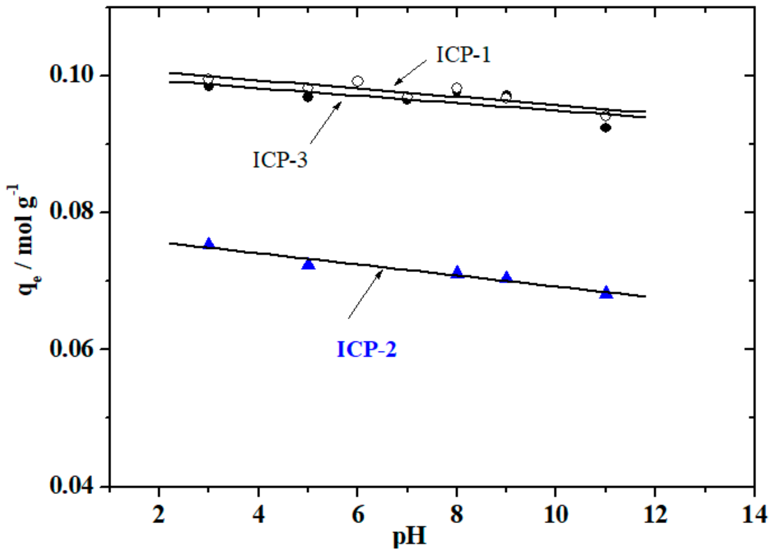

Figure S5. Effect of pH on the equilibrium adsorption of AB25 over nanocomposites ICP 1-3 at 25 °C, (ICP 1-3: 2.0 g/L), and [AB25] = 2 x 10^{-4} mol/L.

Isotherm models

Freundlich isotherm: Freundlich isotherm model is written as [3]:

$$\ln q_e = \ln K_F + (1/n) \ln C_e \quad (9)$$

K_F is the adsorption capacity and $1/n$ is Freundlich constant related to adsorption intensity. The linearity of ln q_e against ln C_e approves the rationality of Freundlich model.

Langmuir isotherm: The Langmuir isotherm [3] describes the monolayer sorption process and can be expressed as:

$$C_e/q_e = (1/q_{max} K_L) + C_e/q_{max} \quad (10)$$

q_{max} represents the maximum loading adsorption capacity and K_L is Langmuir adsorption constant linked with the adsorption energy. The values of q_{max} and k_L were obtained from its linearized form.

Dubinin-Radushkevich isotherm: Dubinin-Radushkevich equation postulates a constant sorption potential (3). Its linear form is given by

$$\ln q_e = \ln Q_m - B\,\varepsilon^2 \qquad (11)$$

$$\varepsilon = RT \ln (1+1/C_e) \qquad (12)$$

where ε is the Polanyi potential, Q_m is the monolayer capacity (mol g^{-1}), *B* is the constant correlated to the energy of adsorption ($mol^2\ J^{-2}$). The values of Q_m and *B* were calculated from the intercept and slope of the plot ln q_e vs. ε^2, respectively. The mean sorption energy, *E* is elaborated from the Eq. [3]

$$E = 1/(-2B)^{0.5} \qquad (13)$$

References

1. Elsherbiny, A.S.; El-Hefnawy, M.E.; Gemeay, A.H. Linker impact on the adsorption capacity of polyaspartate/montmorillonite composites towards methyl blue removal. *Chem. Eng. J.* ***2017****, 315, 142–151.*
2. Elsherbiny, A.S.; Salem, M.A.; Ismail, A.A. Influence of the alkyl chain length of cyanine dyes on their adsorption by Na+-montmorillonite from aqueous solutions. *Chem. Eng. J.* ***2012****, 200–202, 283–290.*
3. Gemeay A.H.; keshta B.E.; El-Sharkawy R. G.; Zaki A.B. Chemical insight into the adsorption of reactive wool dyes onto amine-functionalized magnetite/silica core-shell from industrial wastewaters. Environmental Science and Pollution Research 2020, 27, 32341–32358

© 2020 by the authors. Submitted for possible open access publication under the terms and conditions of the Creative Commons Attribution (CC BY) license (http://creativecommons.org/licenses/by/4.0/).
